# Value of oesophagoscopy and bronchoscopy in diagnosis of synchronous malignancies in patients with head and neck squamous cell carcinomas

**DOI:** 10.1186/s12885-020-07681-9

**Published:** 2020-12-01

**Authors:** Shi Yeung Ho, Raymond King Yin Tsang

**Affiliations:** 1 Department of Ear, Nose and Throat, Queen Mary Hospital, 102 Pokfulam Road, Hong Kong; 2 Division of Otorhinolaryngology, Department of Surgery, The University of Hong Kong, Queen Mary Hospital, 102 Pokfulam Road, Hong Kong

**Keywords:** Oesophagoscopy, Bronchoscopy, Head and neck squamous cell carcinomas, Synchronous malignancies, Field cancerization, Trans-nasal flexible endoscopy

## Abstract

**Background:**

Routine screening of patients with head and neck squamous cell carcinomas (HNSCCs) for synchronous malignancies using oesophagoscopy and bronchoscopy had been controversial. The aim of this study is therefore to find out the rate of synchronous malignancies in patients with primary HNSCCs, the risk factors for its occurrence and the effectiveness of oesophagoscopy and bronchoscopy from a 10-year experience in a single centre.

**Methods:**

A retrospective review of medical records was conducted from July 2008 to June 2018 in a tertiary referral centre in Hong Kong. All patients with newly diagnosed HNSCCs were screened with oesophagoscopy and bronchoscopy at the time of diagnosis and therefore all patients were included in the study. The incidence of synchronous malignancies along the aerodigestive tract and the yield of oesophagoscopy and bronchoscopy were studied.

**Results:**

Of the 702 patients included in the study, the overall rate of synchronous malignancies was 8.3% (58/702), with the rate of synchronous oesophageal and lung malignancies being 5.8% (41/702) and 0.85% (6/702) respectively. Fourteen out of the 41 oesophageal malignancies were only detectable with oesophagoscopy. Only one of the synchronous lung malignancies was detectable by bronchoscopy. Risk factors for synchronous malignancies include male gender, smokers, drinkers and primary hypopharyngeal cancer.

**Conclusions:**

Oesophagoscopy is essential for detecting synchronous oesophageal malignancies in patients with HNSCCs especially in male patients, smokers and drinkers, and it is most valuable in primary hypopharyngeal cancer patients among all primary subsites. Bronchoscopy had a low yield for synchronous lung malignancies and can be potentially replaced by imaging techniques.

## Background

Synchronous malignancies occurring along the upper aerodigestive tract was originally discovered by Billroth [1] and Slaughter [2], with the latter proposing the theory of ‘field cancerization’. The theory of ‘field cancerization’ suggested that with exposure to tobacco and alcohol, synchronous multifocal malignancies can occur in oesophagus and lung for patients with primary head and neck squamous cell carcinomas (HNSCCs). The rate of occurrence of such synchronous malignancies can be as high as 23% [3–6] and their overall survival can be 50% worse when compared with first primaries. Oesophagoscopy and bronchoscopy had been the procedure of choice for screening synchronous malignancies along the upper aerodigestive tract [7–9]. Debates however existed on whether the routine use of them were warranted as there were concerns over risks and cost of procedure and increasing waiting time before start of treatment [10–13]. The aim of this study is therefore to find out the rate of such synchronous malignancies in patients with primary HNSCCs, the risk factors for its occurrence and the effectiveness of oesophagoscopy and bronchoscopy from a 10-year experience in a single centre.

## Method

A retrospective review of electronic medical records was conducted on consecutive patients with newly diagnosed and untreated HNSCCs in a tertiary referral centre (Queen Mary Hospital, Hong Kong). In our centre, newly diagnosed HNSCCs patients were screened with oesophagoscopy and bronchoscopy at the time of diagnosis and therefore all patients were included in the study. The endoscopic procedure was done in outpatient setting under local anesthesia and using trans-nasal flexible endoscopy. With the trans-nasal flexible endoscopy, the nasopharyngeal, oropharyngeal, laryngeal, and pharyngeal areas will be inspected, following by oesophagoscopy (for oesophagus down to oesophageal gastric junction) and bronchoscopy (for tracheobronchial tract).

Synchronous malignancies were defined as malignancies that were detected during the initial workup upon the diagnosis of the primary malignancy at other sites in the upper aerodigestive tract. Only squamous cell carcinoma histological subtype of synchronous malignancies in oesophagus and lung were included. Exclusion criteria included HNSCCs arising from non-aerodigestive tract region in head and neck region (ear, skin, salivary glands, lacrimal glands), tumors with non-squamous cell carcinomas histology, patient with history of nasopharyngeal carcinomas, patients with previous history of irradiation and patients of non-East Asian ethnicity. Information regarding patient’s demographics, subsites and staging of primary and synchronous malignancies, pickup rate by other means of investigations like PET-CT (Positron emission tomography- computed tomography scan), survival statistics (disease free survival and overall survival) will be recorded and studied. Staging of primary and synchronous malignancies was done in accordance to the eighth edition of the AJCC Cancer Staging Manual (TNM classification from the American Joint Committee on Cancer [AJCC]).

All data was input into Excel spreadsheets (Microsoft Excel for Mac 2013 version 15; Microsoft Corporation, Redmond, Washington). Data analysis was done with SPSS version 24.0 software (IBM, Armonk, New York). Differences in means for normally distributed variables was assessed with 2-tailed unpaired t test. Differences in means for non-normally distributed variables was assessed with Mann-Whitney U test. *P* value was set at < 0.05 to be statistically significant.

## Results

Table 1 summarizes the characteristics of the cohort of patients. There were 702 consecutive patients with newly diagnosed and untreated HNSCCs with oesophagoscopy and bronchoscopy done at primary diagnosis in the inclusion period. There were 522 male and 180 female patients. The mean age of the patients was 63 years old (range, 25–94 years old). There were 393 patients (56%) who were active or ex-smokers, 209 patients (29.8%) who were drinkers or ex-drinkers and 182 patients (25.9%) who were smokers and drinkers.

**Table 1:** Patient characteristics, sites and staging of primary malignancies

Parameter	Number of patients
Mean Age at presentation (years ± SD)	63 ± 13.9
Gender (*n*, %)	
-Male	522 (74.4)
-Female	180 (25.6)
Active or ex-smokers (*n*, %)	
-Yes	393 (56)
-No	309 (44)
Active or ex-drinkers (*n*, %)	
-Yes	209 (29.8)
-No	493 (70.2)
Sites of primary malignancies (*n*, %)	
-Oral cavity	356 (50.7)
-Larynx	126 (17.9)
-Hypopharynx	110 (15.7)
-Oropharynx	74 (10.5)
-Paranasal sinuses and nasal cavity	33 (4.7)
Stages of primary malignancies (*n*, %)	
-Stage 1	162 (23.3)
-Stage 2	92 (13.1)
-Stage 3	89 (12.7)
-Stage 4	357 (50.9)

Majority of sites of primary malignancies were in the oral cavity (356 patients, 50.7%), followed by laryngeal (126 patients, 17.9%), hypopharyngeal (110 patients, 15.7%), oropharyngeal (74 patients, 10.5%) and paranasal sinuses and nasal cavity cancers (33 patients, 4.7%). For staging, most of the patients were having stage 4 disease on presentation (357 patients, 50.9%), whereas there were 162 (23.2%), 92 (13.1%) and 89 (12.7%) patients with stage 1,2 and 3 disease respectively.

**Fig. 1:**
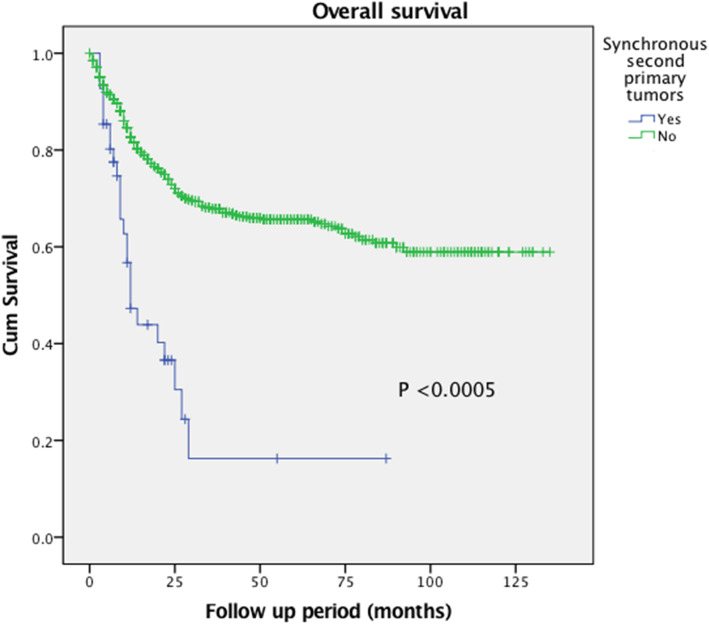
Kaplan-Meier survival curve. Patients with synchronous malignancies associated with significantly better overall survival when comparing with patients with single primary malignancies

**Fig. 2:**
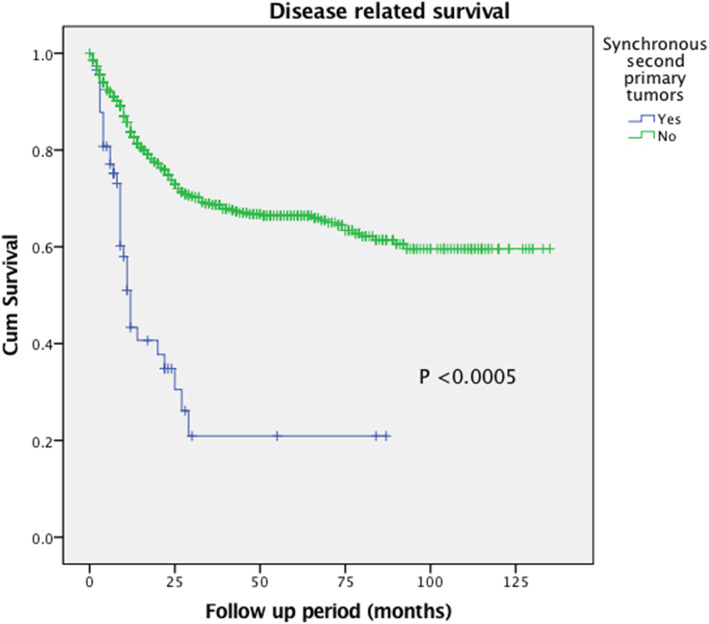
Kaplan-Meier survival curve. Patients with synchronous malignancies associated with significantly better disease-free survival when comparing with patients with single primary malignancies

The rate of synchronous malignancies was 8.3% (58/702 patients). Figures 1 and 2 shows the Kaplan-Meier survival curve showing overall survival and disease-free survival of the cohort. When comparing between patients with synchronous malignancies and patients with no synchronous malignancies (single primary malignancy), the one-year and three-year overall survival (OS) rate were significantly lower in the former group than the latter (one-year OS 40.8% vs 83.25%, *P* < 0.0005; three-year OS 6.8% vs 54.0%, *P* < 0.0005). The same would apply for disease free survival (DFS) rate (one-year DFS 24.5% vs 68.8%, *P* < 0.0005; three-year DFS 6.8% vs 50.1%, *P* < 0.0005).

Characteristics of synchronous malignancies were delineated in Table 2. Among all primary subsites, hypopharynx carried the highest rate of synchronous malignancies at 27.3% (30/110 patients). There was a relatively low incidence of synchronous malignancies in other primary subsites. In terms of sites of synchronous malignancies, most of them occurred in oesophagus (41/58 patients, 73.2%), followed by sites other than the aerodigestive tract (9/58 patients, 15.5%), lung (5/58 patients, 8.9%), head and neck (3/58 patients, 5.4%).

**Table 2:** Characteristics of synchronous malignancies and synchronous oesophageal malignancies

Parameters	Number of patients
Sites of synchronous malignancies (*n*, % among all synchronous malignancies)	
-Oesophagus	41 (73.2)
-Lung	5 (8.9)
-Head and neck	3 (5.4)
-Other sites *	9 (15.5)
Sites of primary malignancies giving rise to synchronous malignancies (*n*, % among total number of patients with the primary malignancy)	
-Hypopharynx	30 (27.3)
-Oral cavity	14 (3.9)
-Larynx	8 (6.3)
-Oropharynx	4 (5.3)
-Paranasal sinuses and nasal cavity	1 (3)
Rate of synchronous oesophageal malignancies (*n*, %)	41 (5.8)
Distribution in oesophagus: (*n*, % among all synchronous oesophageal malignancies)	
-Upper 1/3 of oesophagus	14 (34.1)
-Middle 1/3 of oesophagus	11 (26.9)
-Lower 1/3 of oesophagus	16 (39)
Sites of primary malignancies giving rise to synchronous oesophageal malignancies (*n*, % among total number of patients with the primary malignancy)	
-Hypopharynx	25 (22.7)
-Oral cavity	8 (2.2)
-Larynx	4 (3.2)
-Oropharynx	4 (5.4)
-Paranasal sinuses and nasal cavity	0 (0)
Stages of synchronous primary oesophageal malignancies (*n*, %)	
-Stage 1	22 (53.7)
-Stage 2	4 (9.8)
-Stage 3	10 (24.4)
-Stage 4	5 (12.2)
Risk factors for synchronous oesophageal malignancies (Odds ratio)	
-Male gender	14.89
-Smoker status	3.33
-Drinker status	26.14
-Primary hypopharyngeal cancer	10.59

^*^Other sites (number): prostate (4), liver (2), sigmoid (1), thyroid (1), stomach (1)

There were three synchronous malignancies in the head and neck region. Amongst them, two were detectable on clinical examination (one at tongue from a primary hypopharyngeal cancer and another at lower alveolus from a primary cancer at maxillary sinus). The remaining one was at tongue base from a primary retromolar trigone cancer. It was non-suspicious at initial endoscopy but showed increase uptake when working up with PET-CT and therefore biopsy was performed to confirm the malignancy.

The rate of synchronous oesophageal malignancies was 5.8% (41/702 patients). They were distributed in upper 1/3 of oesophagus (14/41, 34.1%), middle 1/3 of oesophagus (11/41, 26.9%) and lower 1/3 of oesophagus (16/41, 39%). The majority of the synchronous oesophageal malignancies were suffering from primary hypopharyngeal cancers (25/41 patients, 61.0%), following by oral cavity cancers (8/41 patients, 19.5%), laryngeal cancers (4/41 patients, 9.8%) and oropharyngeal cancers (4/41 patients, 9.8%). Most of the synchronous oesophageal malignancies detected were early diseases, with 53.7% of them having stage 1 disease. Risk factors for synchronous oesophageal malignancies included male gender (Odds ratio OR 14.89, 95% CI: 2.03–109.08), smoker status (OR 3.33, 95% CI: 1.51–7.30), drinker status (OR 26.14, 95% CI: 9.18–74.40) and primary hypopharyngeal cancer (OR 10.59, 95% CI: 5.43–20.64). Out of the 41 patients with synchronous oesophageal malignancies, 14 of them (34%) were only detectable with oesophagoscopy and not by other means of investigation including CT scans of the thorax and PET-CT scans. Therefore, the exclusive detection rate for oesophagoscopy among all subjects was 2.0% (14/702 patients). Four of these 14 patients were asymptomatic., 13 of them were having stage 1 disease and only 1 of them was having a stage 3 disease. The overall survival and disease-free survival of patients with oesophageal malignancies exclusively detectable with oesophagoscopy were significantly better when comparing with other synchronous oesophageal malignancies not exclusively detected by oesophagoscopy (*P* = 0.028).

The rate of synchronous lung malignancies was 0.7% (6/702 patients). Among these 6 patients, 4 of them were detected on chest x-ray, one by PET-CT and one by bronchoscopy. All 6 of these synchronous lung malignancies were detectable by PET-CT. Therefore, the detection rate by bronchoscopy was 16.7% whereas the exclusive detection rate by bronchoscopy is 0%, in comparison with the 100% detection rate by PET-CT.

No complications of oesophageal or bronchial perforation occurred amongst all patients in the study period. The median follow-up period for the cohort of patients was 23 months (Range: 0–120 months).

## Discussion

The role of oesophagoscopy in HNSCCs had been debatable in the literature. Amongst North American studies, the rate of synchronous malignancies in oesophagus ranged from 0 to 8% in most studies in the 1980s. However, in a review by Mcgarey et al. [14], there had not been a single synchronous oesophageal malignancy detected on staging oesophagoscopy for patients with HNSCCs since 2000 in North American studies. In contrast, most studies from Taiwan and Hong Kong however had found significant rates of synchronous oesophageal malignancies among patients with HNSCCs. Chow et al. [15] reported a rate of 10% for clinically important oesophageal lesions found among 118 HNSCCs patients undergoing oesophagoscopy for workup. Chung et al. [6] and Huang et al. [16] reported 23.3 and 14.8% synchronous oesophageal malignancies among patients with HNSCCs respectively in their studies. These findings could be explained by the relatively higher prevalence rate of oesophageal malignancies in Asia [17] and the high prevalence of habits of chewing betel nuts in Taiwan [16, 18], which is a common carcinogen for both oesophageal malignancies and HNSCCs.

In our study, the rate of occurrence of synchronous oesophageal malignancies was 5.8% (41/702) and out of these 41 lesions, 14 of them were only detectable by oesophagoscopy, i.e. these malignancies would be otherwise missed by other means of investigation (e.g. CT/PET-CT). Four of these 14 patients were asymptomatic. In addition, most of these malignancies (13/14) found were of early stage (stage 1 cancers). The overall survival and disease-free survival of patients with malignancies exclusively detectable with oesophagoscopy were significantly better when comparing with other synchronous oesophageal malignancies not exclusively detectable with oesophagoscopy (*P* = 0.028). This had highlighted the importance of staging oesophagoscopy in screening out synchronous oesophageal malignancies. It is also effective in screening out early stage disease and potentially benefit patient’s survival.

In our study, the risk factors associated with development of synchronous oesophageal malignancies included male gender, smoker/drinker status and primary hypopharyngeal cancer. Anatomically, the hypopharynx and oesophagus are structures connected continuously and there had been proven association between malignancies from both structures [16, 18–21]. Huang et al. [16] evaluated 248 patients with hypopharyngeal cancers prospectively and reported a rate of 14.8% of synchronous oesophageal cancers and 9.4% of oesophageal dysplasia among the patients. The French ENT society concluded in their 2012 guideline that oesophagoscopy was indicated as staging procedure for patients with hypopharyngeal cancers [22].

As the field cancerization theory [2] suggested, alcohol consumption and tobacco use are risk factors for development of synchronous malignancies in the upper aerodigestive tract. Lower rates of synchronous malignancy development among non-smokers or non-drinkers was found in studies [23, 24]. Alcohol drinking was reported to be associated with increased risk of oesophageal malignancy in a study for patients with HNSCCs [6] (OR:5.90, *P* = 0.020), as well as an independent risk factor for development of oesophageal cancerous or dysplastic lesions in hypopharyngeal cancer patients [16] (OR: 6.95, *P* < 0.05). The French ENT society suggested that oesophagoscopy should be indicated for patients with chronic alcohol intoxication as it increased the risk of development of synchronous oesophageal malignancies [22].

Bronchoscopy had been less utilized as a routine screening procedure for synchronous lung malignancies in patients with HNSCCs when comparing with oesophagoscopy. This could be due to the availability of other non-invasive means of screening modalities such as chest x-ray, CT scan of the thorax [25] and PET-CT imaging. There was also a low yield of bronchoscopy in the literature as a screening modality for HNSCCs patients, ranging from 0 to 1% [21]. In our study, the rate of synchronous lung malignancies was at 0.6%, comparable with the literature. Amongst them, non-of them is exclusively detectable with bronchoscopy and all of them can be detectable on PET-CT, suggesting that bronchoscopy can be potentially replaced by other investigative modalities.

## Conclusion

Oesophagoscopy is recommended for screening synchronous oesophageal malignancies in patients with HNSCCs, in particular for patients who were male patients, smokers and drinkers, and it is most valuable in primary hypopharyngeal cancer patients among all primary subsites. Part of these synchronous oesophageal malignancies are also only detectable with oesophagoscopy and can potentially affect the survival of patients. Due to the low yield of bronchoscopy and can be potentially replaced by other means of investigation, it is not recommended as a screening procedure for patients with HNSCCs. Complication rate of oesophagoscopy and bronchoscopy is extremely low and the procedure is well tolerated using trans-nasal flexible endoscopy.

## Data Availability

The datasets used and/or analysed during the current study are available from the corresponding author on reasonable request.

## Abbreviations

HNSCCs: Head and neck squamous cell carcinomas
PET-CT: Positron emission tomography- Computed tomography
CT scan: Computed tomography scan
OS: Overall survival
DFS: Disease free survival
OR: Odds ratio
AJCC: American Joint Committee on Cancer
